# Ileocecal Intussusception and Obstruction Secondary to Metastatic Melanoma: A Case Report

**DOI:** 10.7759/cureus.46036

**Published:** 2023-09-26

**Authors:** Kai Fu, Brittany Montesino, Rupa Seetharamaiah

**Affiliations:** 1 General Surgery, Florida International University, Herbert Wertheim College of Medicine, Miami, USA; 2 Surgery, Florida International University, Herbert Wertheim College of Medicine, Miami, USA; 3 Surgery, Baptist Hospital of Miami, Miami, USA

**Keywords:** ileocolic intussusception, bowel obstruction, malignant melanoma metastasis, malignant melanoma, ileocecal intussusception, small bowel obstruction, chemotherapy, metastasis, melanoma, intussusception

## Abstract

Intussusception is an uncommon cause of bowel obstruction in adults. Most cases are associated with a pathologic lead point, commonly attributable to benign or malignant tumors. Malignant skin melanoma can metastasize to the gastrointestinal tract and lead to significant morbidity and mortality if left undiagnosed or untreated. In this article, we present the case of a 43-year-old Hispanic female with a history of stage III melanoma on her neck removed four years ago who presented with three weeks of lower abdominal pain, nausea, and vomiting. Abdominal and pelvic imaging showed a high-grade small bowel obstruction with a transition point at the mid-ileum. Diagnostic laparoscopy confirmed an ileocecal intussusception secondary to a 5 cm mass at the lead point. The patient underwent successful resection of the ileum 5 cm from the intussusception and the ascending colon due to the high risk of malignancy. Pathology of the mass was found to be malignant melanoma, but the resected lymph nodes and omentum did not contain any malignancy. The patient tolerated the procedure well and is currently undergoing chemotherapy. This case demonstrates metastatic melanoma as a rare cause of intussusceptions in adults. It emphasizes the importance of considering intussusception when evaluating adult patients with classic lower abdominal pain. Prompt surgical intervention is recommended in suspected cases to address the significant likelihood of malignancy, especially in patients with an oncological history.

## Introduction

Intussusception, the telescoping of a part of the intestine into an adjacent section, is the most common cause of bowel obstructions and abdominal surgical emergencies in children [1]. However, it is considered a rare occurrence in adults, accounting for 1-5% of all bowel obstructions. Therefore, it may not always be immediately considered as a diagnosis during the initial evaluation of adult patients [2]. Intussusception classically presents as acute onset of severe, crampy abdominal pain occurring at 15- to 30-minute intervals with associated nausea and vomiting. As intussusception progresses, venous and lymphatic congestion can occur, which leads to intestinal edema. If left untreated, it can result in bowel ischemia, perforation, and peritonitis. 

In adults, intussusceptions are associated with an identifiable lead point in up to 90% of the cases, with benign or malignant neoplasms being the most common cause [3]. Small bowel intussusceptions are more likely to be caused by a benign etiology, such as the Meckel’s diverticulum, adhesions, lymphoid hyperplasia, and polyps [4]. When the neoplasm is malignant, it is usually due to an already metastasized cancer, such as melanoma. Primary malignancies such as leiomyosarcomas, adenocarcinoma, GIST tumors, carcinoid tumors, and neuroendocrine tumors can also cause small bowel intussusception. In contrast, colonic intussusceptions tend to be more frequently associated with malignancies at the lead point due to an increased risk of adenocarcinomas in the colon [5].

Most cases of malignant melanoma are diagnosed early when surgical removal is curative. However, patients can still develop metastasis after their initial treatment. The small intestine is a common location for gastrointestinal (GI) metastasis due to its rich blood supply [6]. Although one postmortem study found 60% of melanoma patients to have GI tract metastasis during autopsy, less than 5% of patients with melanoma are diagnosed with symptomatic GI metastasis in their lifetime [6-7]. Therefore, reports of metastatic melanoma acting as a lead point for intussusception are still rare. A review of the current literature found case reports of melanoma causing bowel obstruction, but only a few highlighted metastatic melanoma leading to intussusception at the ileocecal valve. In this article, we present a unique case of an adult female patient with a small bowel obstruction caused by an ileocecal intussusception due to metastatic melanoma involving the ileocecal valve. This case report was written in line with the Surgical Case Report (SCARE) Guidelines [8].

## Case presentation

**Figure 1:**
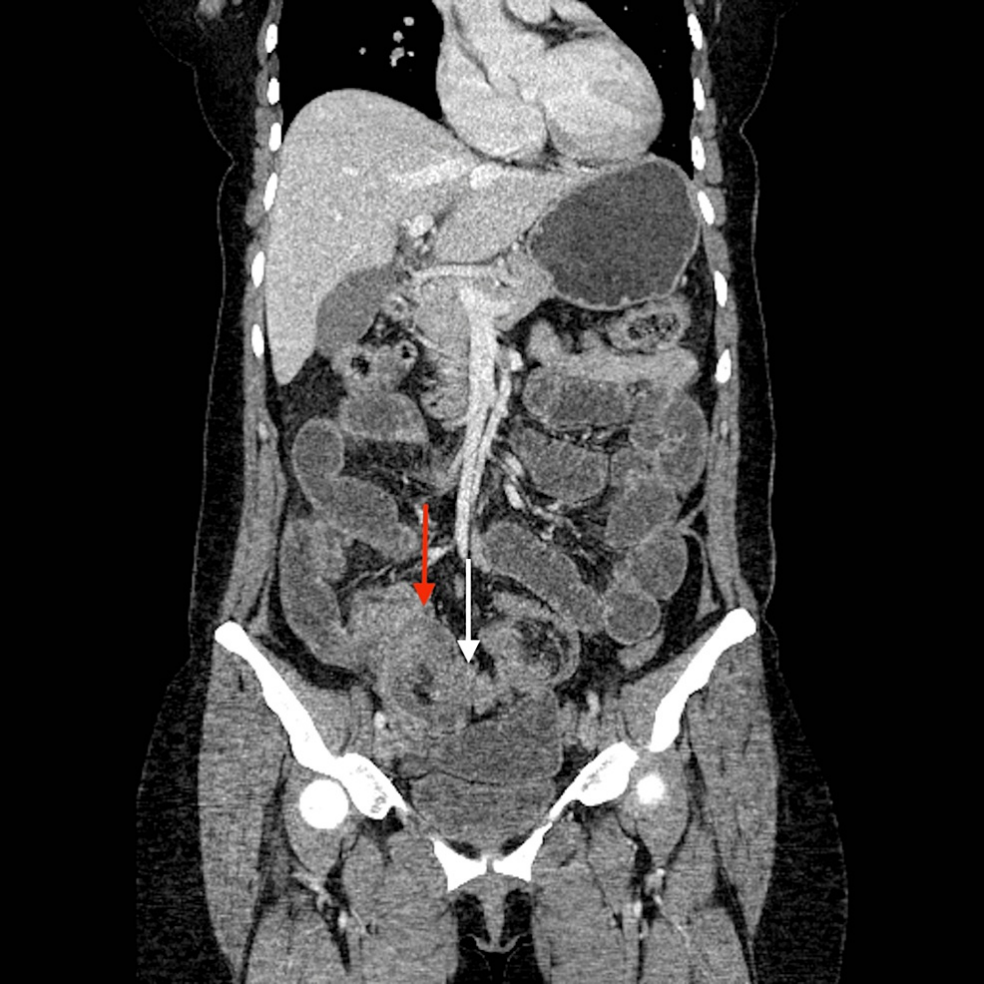
Computerized tomography of the abdomen and pelvis with intravenous contrast, coronal view. Red arrow: intussusception and obstruction; white arrow: abrupt decrease in bowel caliber in the mid-ileum

**Figure 2:**
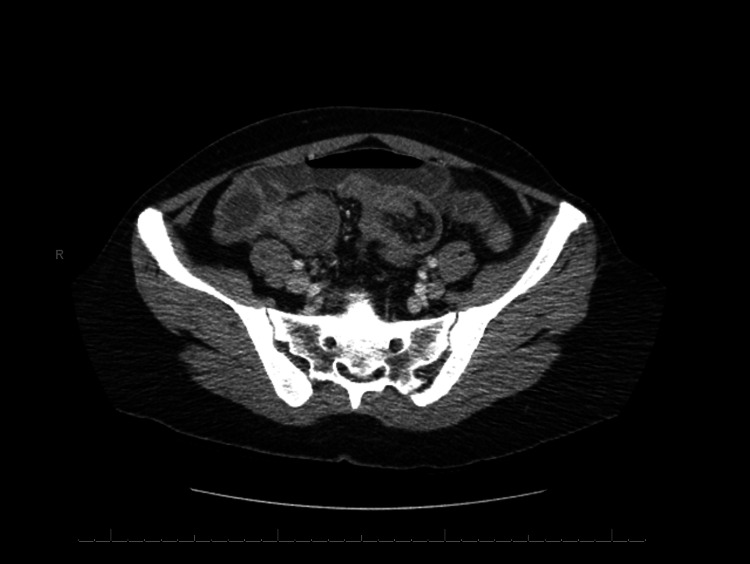
Computerized tomography of the abdomen and pelvis with intravenous contrast, axial view.

A 43-year-old Hispanic female with a history of controlled hypothyroidism and a past surgical history of melanoma removal on the left neck and shoulder four years ago presented to the emergency department (ED) complaining of lower abdominal pain for three weeks with associated nausea and vomiting. The pain was described as intermittently crampy at the onset but progressed to constantly sharp with a moderate intensity three days before presenting to the ED. The patient denied fever, chills, or diarrhea, and she reported that her last bowel movement was on the morning of admission with a normal caliber. The patient was previously diagnosed with pAT3bN1aM0 Stage III melanoma four years ago and was treated with radical neck dissection and chemotherapy with nivolumab for one year. Her follow-up positron emission tomography (PET) scans over the past years were negative except for mildly PET avid lesions in the left neck and right retropectoral and hilar adenopathy without significant standardized uptake value. Her last PET scan one year ago showed a new 8 mm left posterior cervical triangle lymph node with low fluorodeoxyglucose (FDG) uptake, but the lack of significant changes was indicative of a benign process. Her other preventive screenings, including mammogram, colonoscopy, and pap smear completed one year before admission, were all negative. On presentation, she was afebrile with an elevated blood pressure of 150/105 mmHg. Physical examination showed a soft, non-distended abdomen with mild tenderness to palpation of the periumbilical region. Lab results showed slightly elevated hemoglobin at 15.1 g/dL and hematocrit at 45.8%. She had leukocytosis with elevated WBC at 14.79 K/uL with a normal lactic acid level. Her platelet count was also mildly elevated at 484 K/uL. Abdominal and pelvic computerized tomography (CT) scan with intravenous contrast showed diffuse fluid and gaseous distension of the stomach and small bowel loops measuring up to 2.8 cm with an abrupt caliber change in the mid-ileum (Figures 1-2). Those findings were consistent with a high-grade small bowel obstruction (SBO) with a transition point in the mid ileum. The kidney, ureter, and bladder X-ray also showed air-filled loops of the dilated small bowel, supporting the SBO diagnosis. 

**Figure 3:**
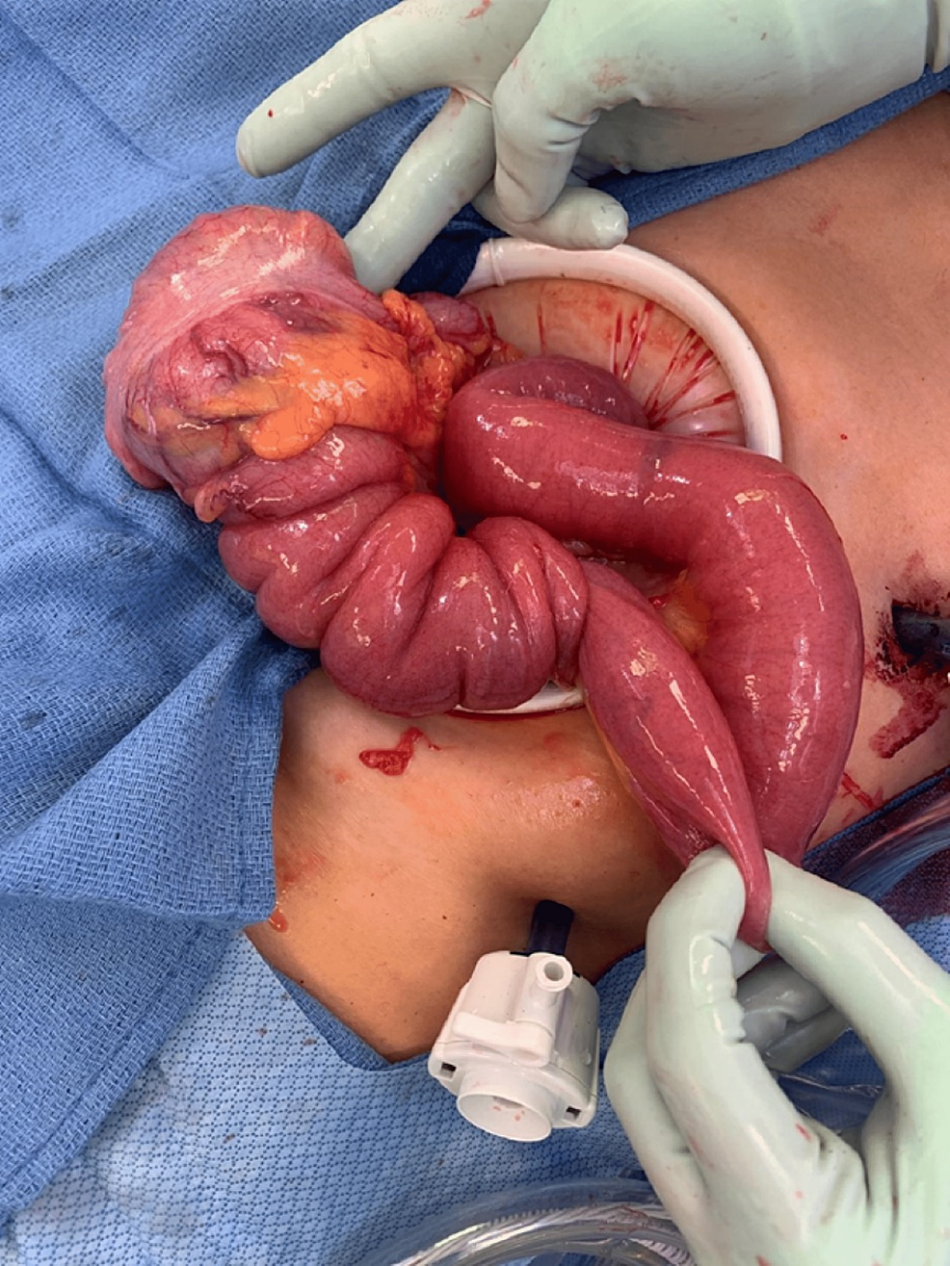
Ileocecal intussusception causing small bowel obstruction.

**Figure 4:**
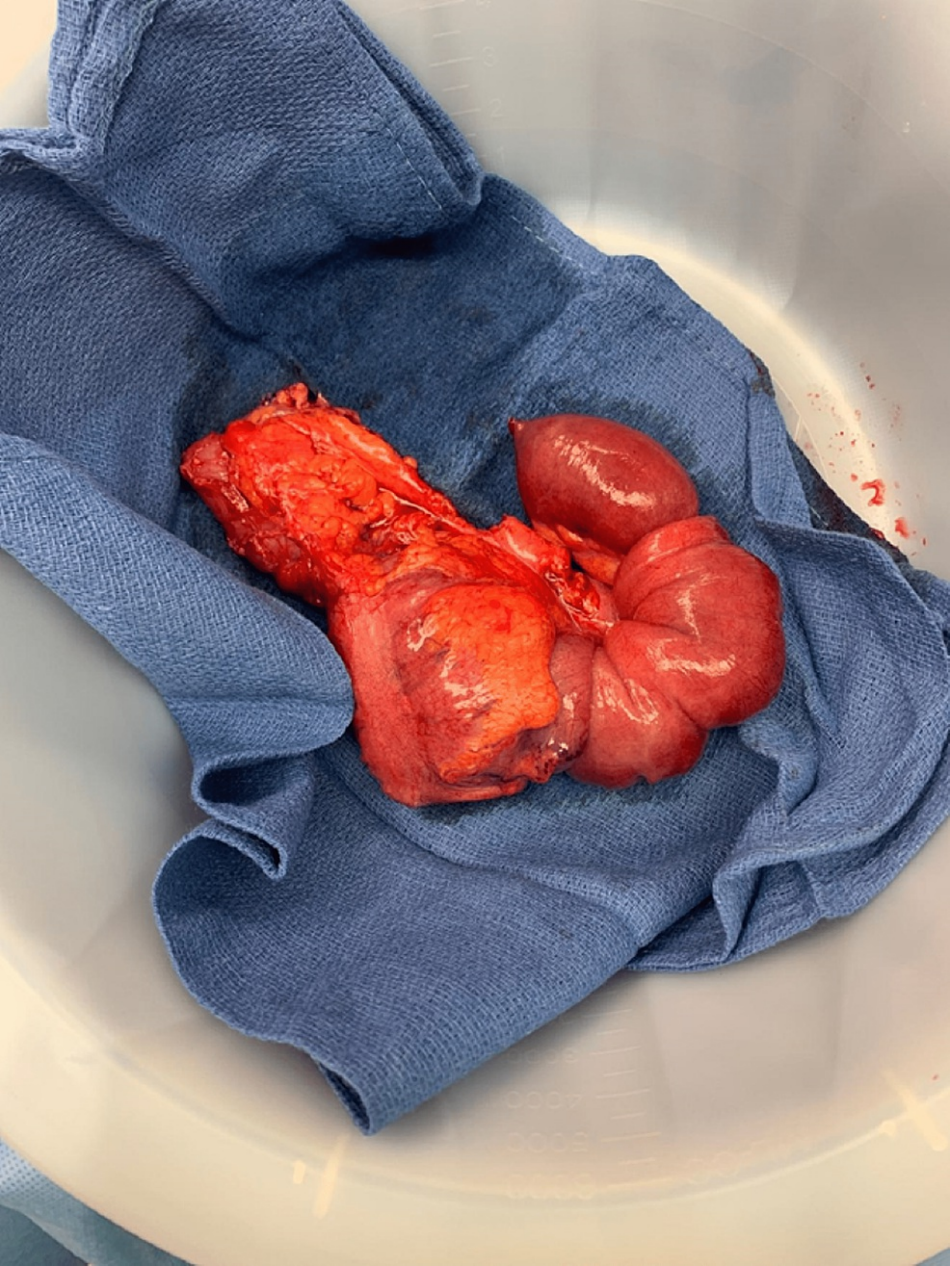
Resected bowel segment containing the intussusception and mass.

**Figure 5:**
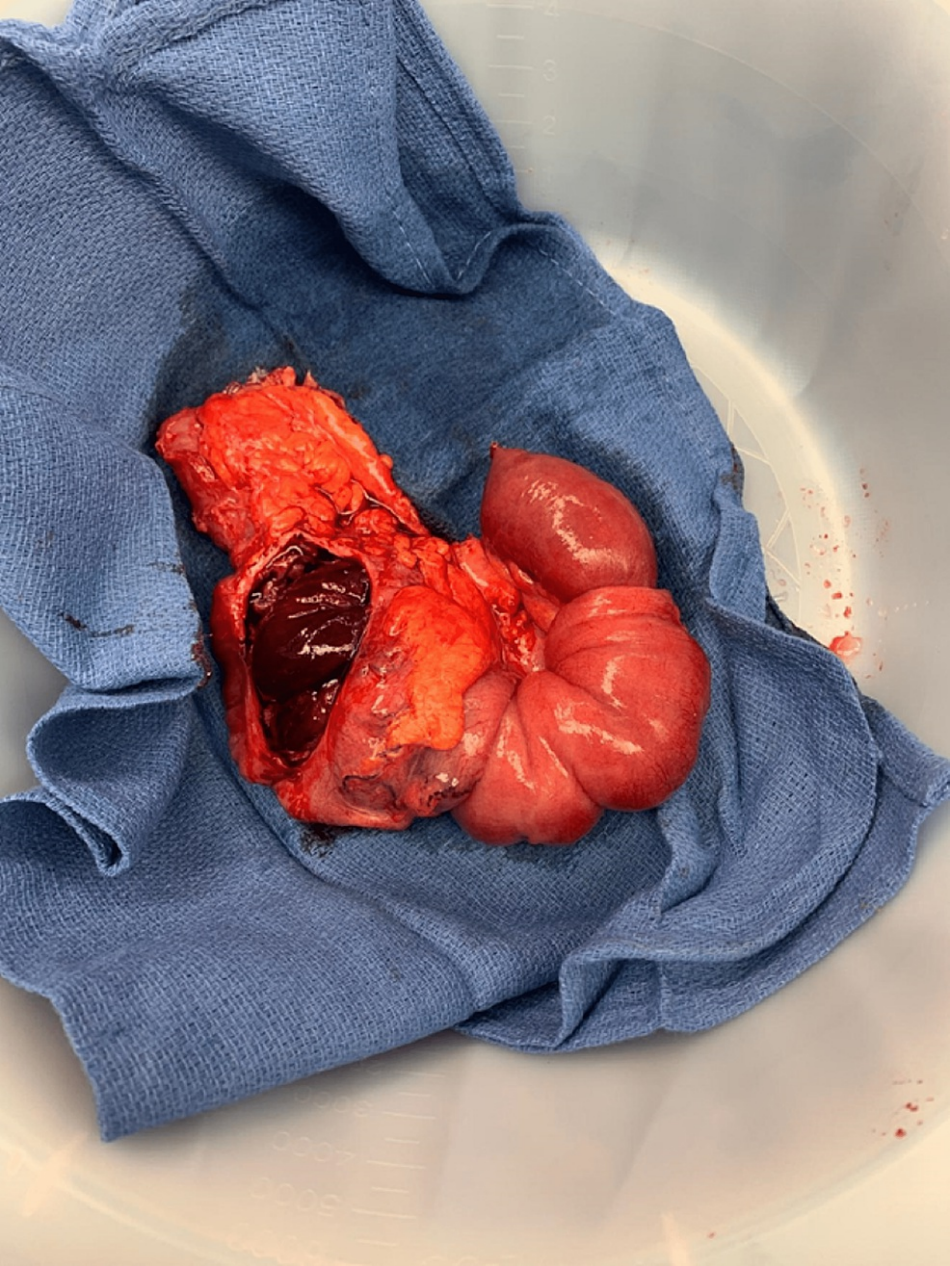
Specimen showing congested small bowel in the lumen of the cecum.

**Figure 6:**
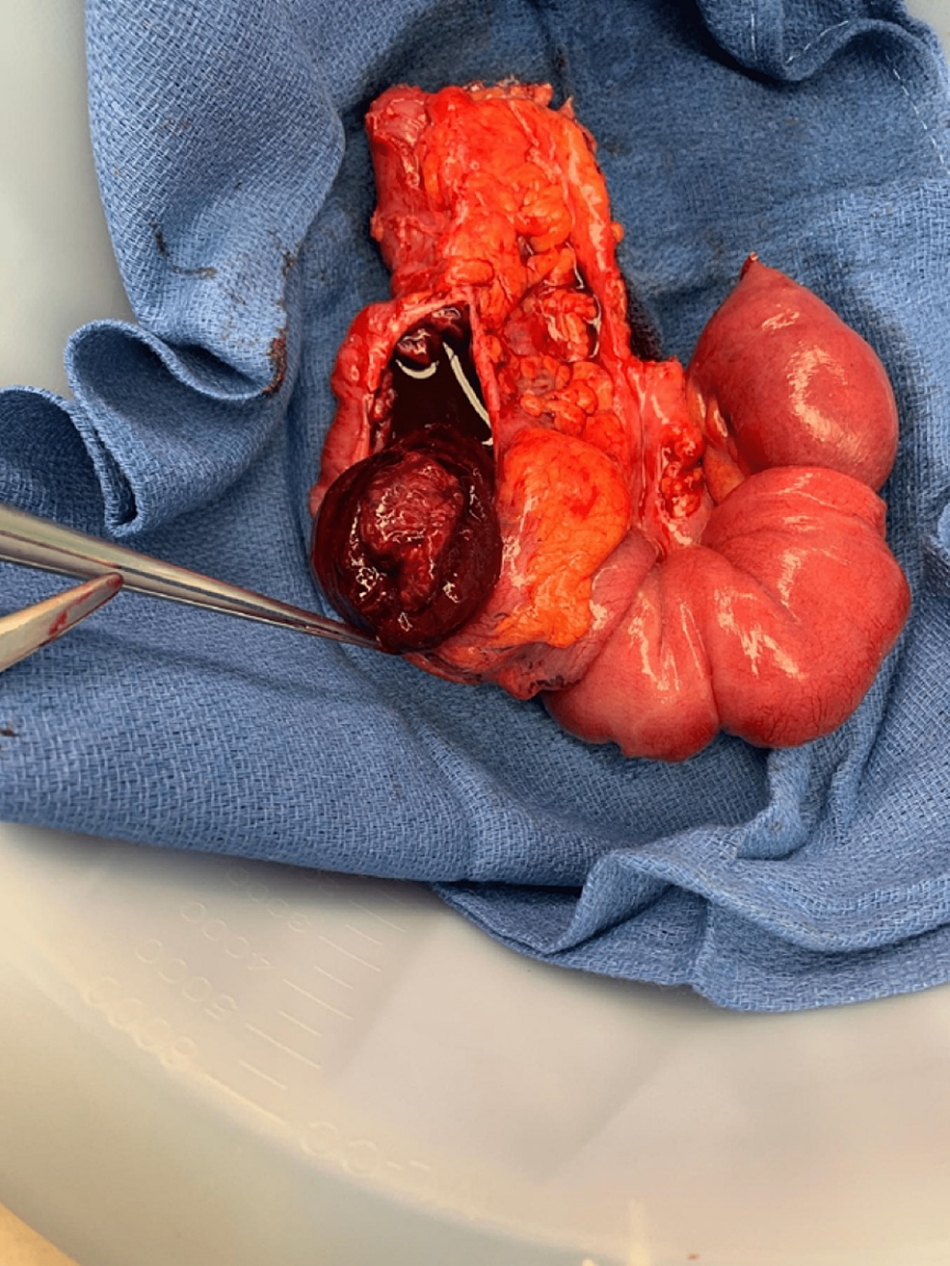
Specimen showing melanoma as the leading point of the intussusception.

**Figure 7:**
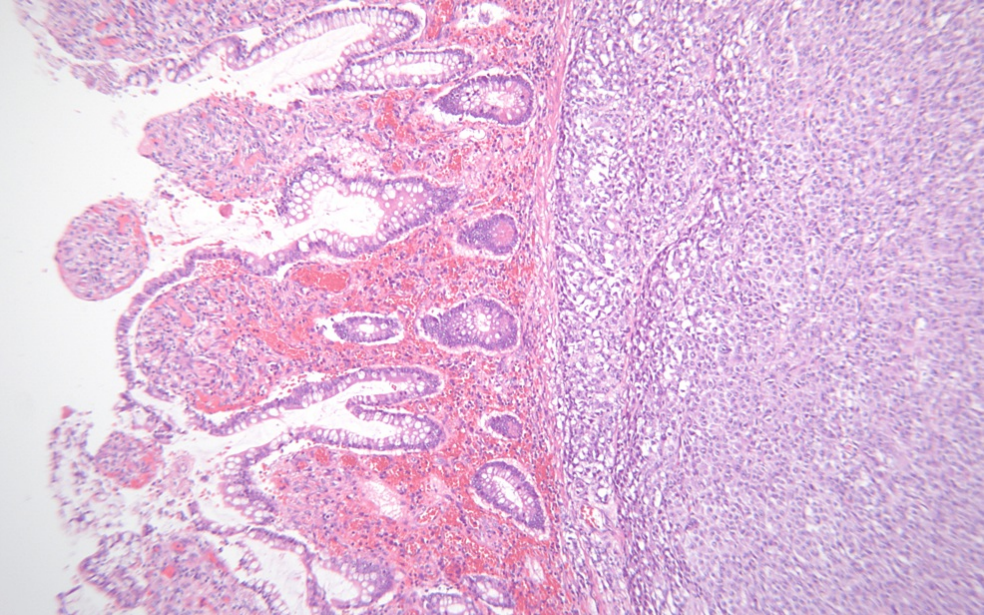
A pleomorphic epithelioid tumor consistent with metastatic melanoma (right) lies immediately beneath small bowel mucosa (left).

**Figure 8:**
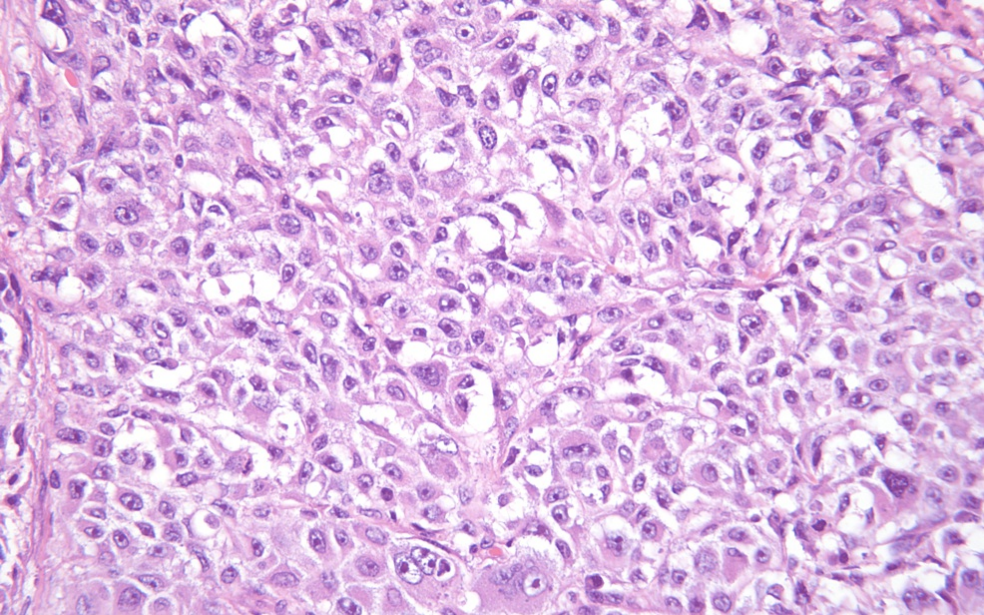
Metastatic melanoma. The lesion consists of large epithelioid cells with irregular nuclear and prominent nucleoli.

General surgery was consulted, and a nasogastric tube with low continuous suction was subsequently placed to decompress the small bowel. After obtaining informed consent, a diagnostic laparoscopy was performed the next day. During the operation, a large, dark, soft tissue mass was identified in the cecum. Upon further inspection, the distal ileum was found to be intussuscepted into the cecum and ascending colon. A laparotomy was performed to better visualize the intussusception (Figure 3). The surgeon performed a hemicolectomy with distal small bowel resection followed by anastomosis of the bowels. The ileum 5 cm from the intussusception and the ascending colon were resected (Figure 4). Macroscopic examination of the excised bowel specimen showed that the terminal ileum was invaginated into the cecum and colon (Figure 5). A 5 cm small bowel mass involving the ileocecal valve was found at the lead point (Figure 6). The small bowel was noted to be congested. A partial omentectomy was performed to complete the operation. Resected bowel, omentum, and lymph nodes were sent to pathology for further evaluation. Pathology of the 5 cm small bowel mass showed metastatic melanoma stained positive for S100, SOX10, and Melan A (Figures 7, 8). The bowel specimen margins were free of tumors, and all 20 resected lymph nodes were benign. Additionally, the resected omentum measuring 20 cm x 14 cm was also negative for malignancy. The patient recovered well from the surgery, and oncology was consulted. Chest CT scan did not show evidence of metastatic disease, and the patient was subsequently discharged home on postoperative day five.

The patient followed up with her general surgeon six weeks after the surgery with excellent recovery. She also followed up with her oncologist, who recommended monthly chemotherapy and remained optimistic regarding her prognosis. At the time of this report, the patient is currently in the fourth month of her chemotherapy treatment, and her follow-up PET/CT scan showed no additional sites of metastasis. The patient gave informed consent for the publication of this case report. 

## Discussion

Malignant melanoma is a common malignancy that metastasizes to the gastrointestinal tract, and the small bowel is the most common location (50-70% of the cases) due to its rich blood supply. Melanoma can also metastasize to other locations of the gastrointestinal tract, including the stomach (27%), large intestine (22%), and the esophagus (5%) [9]. On average, it has been noted to take between three to six years for GI metastasis to occur for melanoma, but the range is quite variable, with some reports being up to 10 years later [10]. The initial diagnosis of melanoma in our patient was four years ago, and despite surgical and chemotherapeutic treatments, she presented with intussusception caused by GI metastasis within the average time period. However, our case is unique because all her follow-up PET/CT scans had been negative, and no other metastatic lesions were found in the postoperative PET scan. Additionally, ileocecal intussusception is exceedingly rare in adults, especially when caused by recurrent cutaneous metastases. 

The management of adults with intussusception is not frequently discussed in literature since intussusception only accounts for 1-5% of all bowel obstructions in adults [2]. The standard of care for intussusception in adults is surgical resection due to the possibility of malignancy as the lead point [2]. One study by Perez et al. found that in asymptomatic patients with incidentally identified intussusception and no evidence of malignancy, nonoperative management, and observation can resolve without surgical intervention [11]. However, resection with curative intent is recommended for symptomatic patients with metastatic melanoma, especially if the metastatic lesion was isolated [11]. Reduction with barium or air enema before the resection is not standardized in adult patients, but it can be considered if the lead point is suspected to be benign, the reduction can be safely done, or the resection is massive without reduction [12-13]. When considering the site of intussusceptions, colonic intussusception is an independent risk factor for malignancy. Colonic intussusceptions should always be resected en bloc without reduction, but reduction may be considered for lesions in the small bowel [14-15]. For our patient, en bloc resection without a primary reduction was chosen due to the patient's history of stage III melanoma and the difficulty in determining whether the lead point was due to a benign process. 

Although our patient received an initial diagnosis of stage III melanoma, she presented with distant metastases four years later, making it a stage IV disease and subject to systemic therapy. The median overall survival of individuals with metastatic melanoma has increased from 11.8 months in 2013 to 21.1 months in 2018 [16], most likely due to many systemic therapies, such as ipilimumab, becoming available through clinical trials in the early 2000s. However, despite these advancements in therapeutic treatments, only 29.8% of the patients with metastasized melanoma between 2011 and 2017 survived for more than five years [17]. Our patient was unaware of her metastatic lesion since she had completed chemotherapy after her initial diagnosis, and follow-up PET scans never showed any clear evidence of recurrence. However, the presence of her symptomatic intussusception led to the incidental finding of metastatic melanoma and resulted in potentially life-saving treatments. Patients with metastatic melanoma who underwent complete and potentially curative metastasectomy for gastrointestinal tract metastases experienced a substantial improvement in overall survival, with a median survival of 64 months [18]. In contrast, patients who opted for palliative and nonsurgical interventions had an average life expectancy of only seven months [18]. This striking difference highlights the significant survival benefits that surgical resection can offer to patients with gastrointestinal metastases from melanoma. However, it is important to acknowledge that surgical resections are still associated with potential risks and limitations, including the possibility of incomplete resection, cancer cell implantation, anastomotic complications, and postoperative infections [19]. Therefore, in order to optimize treatment strategy, it remains imperative to comprehensively assess each patient's unique circumstances and consider individual factors such as the disease extent, overall health status, and feasibility of complete resection.

## Conclusions

This case report illustrates a female patient with a rare presentation of small bowel metastasis from previously treated skin melanoma, leading to a high-grade ileocecal intussusception. Prompt recognition and surgical intervention played a crucial role in the effective management of this patient. This case serves as a pertinent reminder to consider the possibility of intussusception when evaluating adult patients with lower abdominal pain. Physicians should maintain a high index of suspicion in patients with a history of melanoma, given its propensity to metastasize to the gastrointestinal tract. This case also highlights the significant survival benefits of surgical resection due to the high likelihood of malignancy in adult patients with intussusception. Further research is warranted to gain deeper insights into the pathophysiology, optimal treatment strategies, and long-term outcomes of intussusceptions caused by gastrointestinal metastasis. 
